# Psychiatric stigma and discrimination in South Africa: perspectives from key stakeholders

**DOI:** 10.1186/1471-244X-14-191

**Published:** 2014-07-04

**Authors:** Catherine O Egbe, Carrie Brooke-Sumner, Tasneem Kathree, One Selohilwe, Graham Thornicroft, Inge Petersen

**Affiliations:** 1 Psychology, School of Applied Human Sciences, University of KwaZulu-Natal, King George V Avenue, Durban 4041, South Africa; 2 Health Service and Population Research Department, Institute of Psychiatry, King's College, De Crespigny Park, London, UK

**Keywords:** Psychiatric stigma and discrimination, Mental health, Service users, Health care service providers

## Abstract

**Background:**

Stigma and discrimination against people with mental illness remain barriers to help seeking and full recovery for people in need of mental health services. Yet there is scarce research investigating the experiences of psychiatric stigma on mental health service users in low- and middle-income countries (LMICs). The aim of this study was therefore to explore the experiences of psychiatric stigma by service users in order to inform interventions to reduce such stigma and discrimination in one LMIC, namely South Africa.

**Methods:**

Participants comprised a total of 77 adults aged above 18 years, made up of service providers including professional nurses (10), lay counsellors (20), auxiliary social workers (2); and service users (45).

**Results:**

Psychiatric stigma was found to be perpetuated by family members, friends, employers, community members and health care providers. Causes of psychiatric stigma identified included misconceptions about mental illness often leading to delays in help-seeking. Experiencing psychiatric stigma was reported to worsen the health of service users and impede their capacity to lead and recover a normal life.

**Conclusion:**

Media campaigns and interventions to reduce stigma should be designed to address specific stigmatizing behaviours among specific segments of the population. Counselling of families, caregivers and service users should include how to deal with experienced and internalized stigma.

## Background

Stigma and discrimination associated with certain illnesses have remained a global public health concern over the years [1-3]. Treatment stigma has led to major barriers to accessing health care and illness management [4-8]. Stigma has been linked with problems relating to knowledge (ignorance) and attitudes (prejudice) while discrimination has largely been related to behaviour [3]. Different types of stigma exist ranging from public (externalized or experienced stigma) to self-stigma (internalized stigma) [9,10]. Though these types of stigma are interlinked and one can lead to the other, their overall effects on the people with mental illness (PWMIs) can be far reaching. In addition to dealing with their illness, PWMIs have to deal with the social, psychological and economic consequences of psychiatric stigma which can exacerbate low self-esteem, marginalization from society, social isolation, social anxiety, poor social skills, difficulties in securing employment, housing difficulties as well as poor social support, all of which are important for integration into the society [3,5,11-15]. These effects, in turn can lead to strained relationships, depression, low self-esteem, unemployment and be a barrier to accessing health care, etc. [7,16-19]. These consequences are compounded by poor access to health care within a health care system already challenged by widespread inequalities in resources and health personnel [20]. Reducing stigma has hence been identified as an important factor that will improve the lives of those with mental illness [5].

Negative stereotypes by the public and media which portray PWMIs as violent, dangerous, dependent, not fit to get married, psychologically unstable and unfit to work are some of the challenges that these persons have to face from the society in which they live [11]. These stereotypes exist irrespective of the severity or recovery status of patients [21] lending credence to Goffman’s description of stigma as ‘spoiled identity’ [22].

In the light of the fact that persons with mental illness have to grapple with various types of stigma [9], it is important that the health care facilities from where they seek help for their illness should be non-judgmental and value free. The attitude of health care providers is key to ensuring this [23]. Individual beliefs, situational circumstances and personality characteristics have been identified as significant determinants of people’s attitudes towards people with mental illness [23] with society’s conceptualisation of mental disorders having a strong influence on practical professional responses even in the face of information to the contrary [24].

Health care providers are therefore often guilty of treatment stigma [23] of PWMI. With the decentralization of mental health care and its integration into primary health care services in South Africa [25], many general health care providers who have not been exposed to patients with mental disorders, now have to provide health services to users with mental disorders. Previous studies outside of Africa suggest a high level of stigma and discrimination amongst general health care providers as well as mental health care providers [5,3]. Among health professionals, mental health professionals have been found to be less optimistic about prognosis and the long term outcomes for people with mental illness than other health professionals [2,26,27]. This therefore calls for interventions targeted at mental health professionals as well as other health care service providers whose services are vital to the recovery of people with mental illness.

The South African health care system is largely characterized by being organized predominantly to provide acute care as well as inequities between private and public health care that can be attributed to an apartheid past [28,29]. The disease burden of South Africa is characterized by the clashing chronic disease epidemics that the health care system needs to respond to through reorganizing care for integrated chronic disease management. Results from the South African Stress and Health (SASH) Survey show a 75% treatment gap of common mental disorders nationally [30,31]. In addition to other factors that may be responsible for this treatment gap, stigmatization of people with mental illness especially by health care professionals may also be responsible for this treatment gap [4,16,32]. In addition to increasing access to care for people with mental disorders through improved identification and treatment services, there is also a need to address stigmatization of PWMIs to improve service uptake as well as ensure acceptability of services provided [31] especially with the on-going re-organisation of the South African health care delivery sector [33].

In order to inform the development of interventions that may help to reduce stigma in the society, it is important to understand the ways in which PWMIs are stigmatized [19] as well as various stigmatizing agents. This study aimed to explore i) the experience of psychiatric stigma and discrimination by service users with mental illness, at the primary health care level as well as within their families and communities; ii) the perceived causes of stigma and discrimination; iii) the perceived impact of stigma and discrimination on service users; and iv) perceptions on appropriate interventions to address this problem.

Investigating the experiences of stigma and discrimination by service users from their own perspective is especially important because testimonials from service users have been found to be an effective tool for anti-stigma interventions [1]. Unfortunately, there has been little research focusing on the lived experiences of those experiencing stigmatizing attitudes particularly service users [2,16,19,21].

## Methods

### Study design

This study used a qualitative research design using qualitative individual interviews and focus group discussions. It reports on baseline data collected as part of the formative stage of the Programme for Improving Mental health care (PRIME) in South Africa to inform the development of a mental health care plan and accompanying interventions. PRIME is a research consortium which aims to provide evidence on the implementation and scaling up of treatment programmes for priority mental disorders in five low-and middle-income countries across Africa and East Asia [34].

### Study site

The study site was the Dr Kenneth Kaunda District (KKD) in the North West province of South Africa. The KKD is a predominantly urban location with approximately 10% rural population. The population is approximately 632,790 [34]. There are 4 sub-districts and the formative study was conducted in the Matlosana and Tlokwe sub-districts, both urban locations with mixed housing, public transport and public health facilities including regional hospitals, primary health care facilities and a specialist in-patient mental health facility. The North West province has however been identified as one of the under-resourced provinces in South Africa [20].

Kenneth Kaunda District was chosen as the study site with the approval of the South African Department of Health as it is a pilot site for the South African re-engineered primary health care system [35].

#### Participants

A total of eighty-seven (77) participants were purposively sampled to include 32 health care service providers and 45 mental health service users with depression and schizophrenia. This sample size caters for the inclusion of various categories of mental health care service users and health care service providers so as to get a fair representation of the key stake holders involved in mental health care. Service users were all adults recruited from primary health care centres and an NGO servicing people with severe mental illness within the study area. Table 1 presents the demographics of the study participants.

**Table 1:** Sample demographics

	**Depression comorbid with HIV**	**Schizophrenia- service users**	**Maternal depression**	**Health care service providers:**
				**Nurses, Lay counsellors, Aux. Social workers**
**Sample size**	15 service users with depression co-morbid with HIV+	10 service users;	20 women diagnosed with maternal depression	10 nurses;
				20 lay counsellors;
				2 Auxiliary social workers
**Age range**	28 – 53 years	21 – 59 years	21 – 59 years	**Nurses:** 32 – 54 years
				**Lay counsellors:** 23 – 49 years
				**Auxiliary social workers:** 37 and 50 years
**Gender**	All females	6 males; 4 females	All females	**Nurses:** 9 females; 1 male
				**Lay counsellors:** 16 females; 3 males
				**Auxiliary social workers:** 2 females
**Race**	All black Africans	9 black African, 1 white	All black Africans	All black Africans
**Level of education**	1 – None	None-2;	15 – Primary education;	**Nurses**: All professional nurses trained in a 4 year National Diploma in General nursing. Three nurses had received further training for Primary Health Care which made them clinicians. One nurse was trained in HIV management, Management in nursing and had a Diploma in nursing education. One nurse reported being a psychiatric nurse. One nurse was studying towards a degree in nursing and was in her final year.
	7 – Primary education	Primary Education-3;	2 – Secondary education;	
	7- Secondary education	Secondary and Post-Secondary −5	3 – Tertiary education	
				**Lay counsellors:** All of the counsellors had received at least some form of counselling training from various non-governmental organisations including adherence training, HIV counselling, PMTCT and TB.
				**Auxiliary social workers:** Both trained in auxiliary social work. One also trained in paramedics, home based care, and is a trained police reservist.
**Employment status/length of employment**	2 – Employed	Employed-1;	4 – Employed;	**Nurses and lay counsellors:** Work experience ranged from 2 months to 9 years.
	13 – Unemployed	Unemployed-8;	16 – Unemployed	**Auxiliary social workers:** 3 years each.
	1 – Student	Student-1		

### Procedure

Service users (other than those with severe mental illness) were recruited from the waiting rooms of three large primary health care facilities in the study area. Service users with severe mental disorder were recruited in two ways using a convenience sampling approach: (i) through clinic registers held in two primary care clinics, and (ii) through the North West Mental Health Society. Convenience sampling was used for the benefit of finding participants who fall within the scope of the PRIME research project and because of the integration of all categories of patients in PHC in South Africa. All service users with severe mental illness were stable patients receiving chronic medication for schizophrenia/bi-polar disorder. Criteria for inclusion of these service users were: a confirmed diagnosis of schizophrenia/bi-polar disorder, being capable of participating in the interview and being over the age of 18. Their ability to participate in this study was judged by the auxiliary social workers/social workers from the South African mental health society. Other service users were recruited on the basis of meeting the diagnostic criteria for the priority conditions of depression and maternal depression. Exclusion criteria include; not being capable of participating in the interview, not being diagnosed with schizophrenia/bi-polar disorder or depression and being under the age of 18. Recruitment of study participants was done by members of the PRIME-South Africa research group.

The interviewer explained the study to each participant in their preferred language after which they signed the informed consent form as an indication of a voluntary participation in the study. See Table 2 for a summary of the participants and methodology employed. All but one interview were conducted in Setswana (the predominant language in the study site). One interview was conducted in English language.

**Table 2:** Summary of study procedure

	**(Depression comorbid with HIV)**	**(Schizophrenia- Service users; Aux. Social workers)**	**Maternal depression**	**Nurses, Lay counsellors**
**Sample size**	15 service users with depression co-morbid with HIV+	10 service users and 2 Auxiliary social workers	20 women diagnosed with maternal depression	10 nurses; 20 lay counsellors
**Where were they sampled from?**	PHC facility in DRKKD	Clinic and Mental Health Society Klerksdorp	PHC facility in DRKKD	Their respective clinics; Grace Mokhomo, Majara Sephapo, Kanana, Orkney
**Sample description (include eligibility criteria)**	HIV + patients who met the diagnostic criteria for major depressive disorder. Participants over age 18 participants, were not pregnant at the time and had not delivered a baby in the past 5 months. Were diagnosed as HIV + & did not require urgent medical attention	Service users – schizophrenia/bi-polar diagnosis, over 18, able to participate in interview	20 Women over the age of 18 attending postnatal clinics	Purposive volunteer sampling was used.
				Nurses: Professional nurses were requested to do interviews and those available and willing were interviewed
			Services who met the diagnostic criteria for major depressive disorder & whose infant was between 6 weeks and 12 months old	
				Lay counsellors: All lay counsellors in all four clinics were requested to do the focus group interviews. The interviews were done for those who were present on the day of the interview
**Average time period per interview**	50 minutes	45 mins – 1 hr	50 minutes	±45 minutes
**Where was the interview conducted?**	At the facility	Mental Health Society Offices or Clinic	20 At the facility	Nurses and counsellors- onsite, Facility managers- at an eatery
			10 follow-up interviews in the home	
**Informed consent**	The study was explained to the patients and informed consent was obtained	Informed consent forms signed by participants after the study was explained to them	The study was explained to the patient and informed consent was obtained	Consent forms signed by all participants
**Were participants compensated for their time? How much or what was given to them?**	Yes. R50-00 vouchers from a local supermarket	Yes. R35 vouchers from a local supermarket	Yes. R50-00 vouchers from a local supermarket	Participants were not compensated for their time. The facility managers were interviewed over lunch
**Who conducted the interviews?**	A Clinical psychologist	2 Clinical psychologists	2 Clinical psychologists	1 clinical psychologist and 1 research psychologist
**Language the interview was conducted**	Setswana	18 Setswana; 1 English	Setswana	Setswana
**Interpreter used? (Yes/No)**	No	No	No	No
**Procedure for first round analysis**	Guided thematic content analysis was used. Transcripts were analysed using the NVIVO software	Thematic content analysis was used aided by the NVIVO software	Thematic content analysis was used. Transcripts were analysed using the NVIVO software	Thematic content analysis was used aided by the NVIVO software

### Data collection

Four focus group discussions were held with a total of nineteen (19) lay counsellors with each group comprising of between two to seven participants. The other fifty-eight (58) participants were individually interviewed. All interviews lasted between 45 minutes to one hour each. Interviews were recorded using a voice recorder and were then transcribed and translated. Semi-structured interview schedules were used to guide the interview process among the various categories of participants. Health service providers were asked, *inter alia*, about their feelings regarding dealing with PWMIs and whether they have witnessed PWMIs being disrespected, ignored or discriminated against either in the community or in health centres. Service users were asked a variety of questions which included what they thought about their health condition and how their condition affected the way they were treated in their families, communities and in the health facilities. All participants were asked to give suggestions on how attitude towards PWMIs in their families, communities and health centres could be improved.

### Data analysis

Two rounds of data analysis aided by the software NVIVO 10.1 were carried out after the transcription and translation of the interviews. Interviews conducted in Setswana were transcribed and translated to English language. Back translation was done for these data to ensure their validity.

An initial thematic analysis of various segments of the data was conducted by all authors using an ‘a priori’ coding framework based on the interview schedule and themes emerging through an initial familiarisation process. The initial themes generated covered the broad issues on stigma and discrimination including other broader issues being investigated as part of the PRIME-SA project. The data on stigma and discrimination obtained from this first round of analysis was again subjected to thematic analysis by the first author to identify more specific themes and subthemes pertinent to the objectives of this study. Both deductive and inductive approaches were therefore employed in the analysis.

#### Ethical consideration

Permission to conduct the study was obtained from the University of KwaZulu-Natal Research and Ethics Board (ethical clearance number HSS/0880/011), and the North West Provincial department of health. Code names were used to protect the identity of all participants. The confidentiality of information provided was assured the participants in the consent form.

The methodological guidelines for qualitative research reporting (RATS) were adhered to in the reporting of the qualitative study reported in this paper [36].

## Results

### Summary of themes and subthemes

A summary of the themes and subthemes arising from the data is presented in Table 3.

**Table 3:** Summary of results

**Themes**	**Subthemes**
Types and forms of stigma and discrimination	• Internalized stigma
	• Externalized stigma
Experiences of externalized stigma	1. From health professionals and in health facilities
	• General ill-treatment from clinic staff:
	• Avoiding attending to PWMIs and other ill treatment from nurses
	2. From family members
	• Being; denied of food;
	• made fun of;
	• neglected;
	• beaten;
	• tied to a tree
	3. From community members (neighbours, employers and friends)
	• Being; labelled
	• made fun of
	• pushed around
	• denied entrance to shopping outlets
	• made to do filthy jobs
	• denied wages for jobs done
	• lack of support and empathy
Causes of psychiatric stigma	Stigmatizing misconceptions about mental illness
	• Mental illness being a deliberate act
	• PWMIs are aggressive
	• Mental illness is a result of the individual’s weakness
	Traditional explanatory models of mental illness which may lead to delay in seeking help
	• Mental illness caused by witchcraft
	• Mental illness being a sign indicating a call to be a ‘*Sangoma*’
Impact of stigma on service users	• Being unable to lead normal lives
	• Worsened state of health
Interventions to curb psychiatric stigma: participants perspectives	1. Education
	i. Education/awareness raising for:
	• Family members
	• Community members
	• Service users
	• Service providers
	ii. Education methods:
	• Health education
	• Media (pamphlets, TV, radio)
	• Town hall/community meetings
	• Health talks at clinics
	iii. Psycho-education and psychosocial rehabilitation for family members and service users
	2. Acceptance and support by family and community members
	3. Supervision of health care service providers
	4. Integration at health facilities
	5. Sanctions/legal action against agents of discrimination

Results have been categorised into four major themes with their associated subthemes as presented in Table 3. These major themes are; service users’ experiences of stigma and discrimination, causes of psychiatric stigma, impact of stigma and discrimination on service users and suggestions to combat stigma (from the perspectives of the study participants). Results are discussed under their major themes and subthemes.

1. **Experiences of stigma and discrimination**

Results from this section have been grouped according to the different types and forms of stigma and discrimination experienced.

1.1 ***Internalized stigma***

The internalization of the stigmatizing attitudes experienced by PWMIs was a common feature in the reports of service users. The impact on their help-seeking behaviour was reported as follows:

*Something that can prevent people from seeking help…there are people who are afraid, someone knowing that she/he has problems, but they think "what will people say?", that is the thing that can prevent people (from seeking for help). –****Service user (DH17)***

1.2 ***Externalized/experienced stigma***

Results in this section have been grouped according to the places were stigmatization is perpetuated as well as stigmatizing agents which include, health professionals, family members and friends and community members.

1.2.1 *Ill treatment at health facilities and by health facility staff*

While some service users reported being treated well at the clinics, there were reports of stigmatization of service users at the clinics where they seek help. From security personnel attached to health care facilities to clinic staff, there were reports of PWMIs being beaten, shouted at, being made fun of or simply ignored. These experiences can lead to PWMIs being hesitant to seek help.

*Yes. There is the other one who used to come to the clinic and cause a lot of commotion. The security guard who used to work here in the clinic would then beat him up… The security guard who used to work here was a female and the person who suffered from mental problem was a male. She would beat him up and push him out of the clinic premises (saying)… he is not our patient here in the clinic. –****Lay counsellor (LCK3)***

It is interesting to note that both service users and professional nurses reported that PWMIs sometimes get shouted at or made fun of at the clinics as exemplified in the following narrative by a nurse. This behaviour can deter PWMIs from seeking help at the clinics.

*Yes, most people would make fun of mentally ill patients and they would say funny comments such as “this is a lunatic”. We used to experience that at this clinic, it’s not nice at all and this can happen to anybody. –****Nurse (N5)***

Professional nurses were also accused of refusing to give medical attention to PWMIs as explained by a lay counsellor.

*You find that all the professional nurses don’t want to do mental health work. –****Lay counsellor (LCMS6)***

It should be noted that many of the health professionals when asked about whether stigmatizing and discriminatory practices towards PWMIs occur at their facilities were reticent to mention such incidents. They seem to be more comfortable providing examples of such behaviours in families and the communities.

1.2.2 *Ill treatment from family members*

Various experiences of stigmatization and discrimination within the family were reported. These included PWMIs being denied food; laughed at; neglected; beaten and being tied to a tree. Some service users recounted the treatment they got from their family members while service providers testified to having witnessed some of these stigmatizing behaviours either in their homes or around their neighbourhood.

*They tie them up…they would tie her leg to the tree and she won’t be able to walk, and most of the time they wouldn’t bath her, she was dirty. –****Lay counsellor (LCO1)***

*Do you mean my family? They just like saying things like I should remember that I’m a lunatic. That hurts and that’s why I end up crying. –****Service user (S6)***

The lack of care showed by some members of the family of PWMIs seems to pose a challenge to the work of health care service providers as mentioned in the narrative below.

*They also complain that even in their families they also get discriminated and they don’t give them food and that’s why they end up eating in the dustbins. They only bath them when they go to collect their grant. –****Auxiliary social worker (ASW2)***

1.2.3 *Ill treatment from community members*

The experiences of stigma within the community have been presented here under the various stigmatizing agents within the community. These include neighbours/general public, church members, friends and employers.

*From neighbours/ church members, friends and other members of the public:* Reports from service providers and service users indicate that PWMIs suffer a lot of stigmatization and discrimination from the members of their community including children. They are provoked, called names and pushed around because of the state of their mental health. This ill-treatment is not stopped even if PWMIs are stabilized or getting better. One cvparticipant saw this as a problem peculiar with the black race group.

*I’ve seen one woman in town who has a mental illness, people were pushing her around and the more they did that, the more she got aggressive. –****Nurse (N1)***

*Black community has one problem, if a person has a mental illness and gets better, he will always be stuck with a lunatic name....the more he receives teasing he might get stressed out and relapses. –****Auxiliary social worker (ASW2)***

*From employers:* Discrimination from employers was also reported with PWMIs sometimes being inadequately compensated for work done or made to do filthy jobs that other members of the community would normally not want to engage in.

*There are so many people in the community who make them do filthy jobs. –****Auxiliary social worker (ASW1)***

2. **Causes of psychiatric stigma**

Psychiatric stigma stems from a multiplicity of reasons, many of which stem from beliefs about the causes of mental disorders. Some of these misconceptions or traditional beliefs are stigmatizing in themselves, while others result in delay in seeking help for PWMIs. The beliefs presented here include those about depression and schizophrenia.

2.1 ***Stigmatizing misconceptions about mental illness***

Mental illness being a deliberate act

One of the misconceptions about mental illness that emerged is the belief held by community members that people with mental illnesses are deliberately pretending to be sick and were deliberately acting out the symptoms of mental illness they displayed. This perception also contributes to a delay in help seeking. This is explained as follows by a participant.

*There are those who hallucinate and they would say …he is acting that way deliberately and they delay seeking help for that person… –****Auxiliary social worker (ASW2)***

The reason for this misconception may be because apart from their behaviour which sometimes does not conform to the norm in the society especially when they are seriously ill, PWMIs generally look physically well. One service user shared his experience when asked why he thinks people with mental illness are being stigmatized.

*The thing is, they say we are pretending and we are not. –****Service user (S2)***

PWMIs are aggressive

People with severe mental disorders such as psychosis/schizophrenia were also perceived as being aggressive. Although this misconception is based on the behaviour of PWMIs when having a psychotic episode, it was presumed to apply to all persons with mental illness, irrespective of their condition or whether they were symptomatic or asymptomatic. Health care service providers expressed a fear of working with service users with severe mental disorder as a result of this perception.

*I’m scared to work with them because I know crazy people as aggressive people. –****Lay counsellor (LCO2)***

Mental illness being a result of the individual’s weakness

The belief that mental illness is due to a person’s weakness was also prominent. Surprisingly, this view was expressed by two health care workers. This misconception can be both stigmatizing and delay referring service users for specialist care.

*You see that one (schizophrenia)… in my point of view it does not need treatment. It’s just an individual’s weakness. –****Lay counsellor (LCGM1)***

2.2 ***Traditional explanatory models of mental illness which may lead to delay in seeking help***

Mental illness caused by witchcraft

A common understanding of the cause of mental illness in the community was the belief that people with mental illness have been bewitched. Due to this belief system, many families seek care from African traditional healers (*sangomas*) before they visit the clinics. This belief also results in a delay in seeking medical help as expressed in the following narratives. It should be noted that recourse to *sangomas* as the first port of call in seeking for solutions to problems relating to health and general well-being, is not a phenomenon peculiar to the study participants as it is a common phenomenon especially among black South Africans [37,38].

*The belief is that the person would have been bewitched and that’s why the person would be taken to a ‘sangoma’; they are taken there to find out who is it that bewitched them to become mentally ill. –****Lay counsellor (LCK3)***

Mental illness as a sign indicating a call to be a ‘Sangoma’

It was also mentioned that sometimes, the symptoms of mental illness are sometimes taken for a sign indicating a call by the ancestors to take up the role of a traditional healer (a ‘sangoma’) or as being gifted. Seeking help based on early symptoms therefore becomes delayed as the family members would rather go to the traditional healers first.

*Yes until complications…until serious complications because the person would tell someone or a family member that ‘I’m hearing people talking to me’. They would say maybe you are gifted in spiritual issues of traditional healers. Another person would say I’m seeing people here who have already passed on; they also say it’s a gift. –****Nurse (N9)***

3. **Impact of stigma on service users**

Reports from participants indicate two major effects suffered by PWMIs as a result of stigma and discrimination: being unable to lead normal lives; and a worsening state of health of the service user. A worsening state of health could be as a result of the direct consequence of stigmatizing attitude or indirectly due to a delay in seeking help for PWMIs or being given poor treatment as a result of the stigmatizing behaviour of service providers at the clinics. This also impacts on adherence to routine care as PWMIs who are discriminated against will be reluctant to return to the clinic to follow-up on their treatment regimen.

3.1 ***Being unable to lead normal lives:***

Results suggest that PWMIs become home bound as a result of the real and perceived fear of being stigmatized. This makes them unable to live and move around their neighbourhood or carry out normal activities like other members of their community.

*I couldn't get out of the house to fetch some water from the standpipe because the moment you come out of the house, people would be looking at you. If I go to church; I was afraid they would say that she has started to attend church because her life has failed. –****Service user (MD17)***

3.2 ***Worsened state of health:***

Reports from all categories of participants in this study point to the fact that stigmatizing attitudes worsen the mental health of PWMIs. This is evident in the following narrative.

*(They say) “You behave as if you are crazy and you are not.” When they say that, the voices attack me and become louder.... My father is the person who says I sleep too much… I become irritated when he speaks like that. –****Service user (S2)***

4. **Interventions to curb stigma: perspectives from participants**

In this section results on the suggestions made by all the categories of participant as to what they think should be done to address psychiatric stigma are presented. These include education, counselling, acceptance, care and support, training, integration at health facilities, sanctions/legal actions on those who perpetuate discrimination and supervision of health care staff.

4.1 ***Education***

Suggestions on how to use education or raise awareness aimed at curbing psychiatric stigma have been grouped here under two headings; who to educate and suggested means of education. Psycho-education and psychosocial rehabilitation of service users and family members were also suggested.

4.1.1 *Who to educate*

Education of family and community members as well as service users themselves was highlighted as key to reducing stigma. The Family, community members and health care service providers were reported to need education to help them understand what service users are actually going through to motivate them to seek help for the person with mental illness rather than ignoring them or having them beaten or treated with disrespect.

*Psycho-educate their family members about their illness so that they can treat them just like any other normal person.* ***– Nurse (N1)***

It was also suggested that service providers need to be educated on how to handle PWMIs.

*These (health) services need for people to be educated…on how to handle a patient… how to handle people. They need people who are humble…those who know how to talk and handle themselves. –****Service user (DH14)***

In the same vein, it was suggested that mental health service users should be educated especially about the type of mental disorder they may be suffering from. This would enable them understand what they may be going through and would help them cope better as reported by a participant with maternal depression.

*The information about these things is not easily accessible and doesn't reach most of us. For me to know that I am depressed is through reading; there is no one who told me about depression. I like reading so; I am able to search for information on my own. –****Service user (MD13)***

Awareness by community members of the problems faced by PWMIs can enable community members to assist PWMIs to seek help as expressed by one professional nurse.

*Yes, I think if the community could be involved that would make the situation much better. If they know the signs and symptoms they can identify them in the community and send them to the clinic to get help.* ***– Nurse (N5)***

4.1.2 *Educational methods*

Participants also highlighted the methods of education that they perceived would be effective in combating psychiatric stigma. These include; awareness programmes through health talks at the clinic, media outreach through pamphlets, and in the print and electronic media, town hall, church and community meetings.

*We lack information if there were pamphlets being distributed about people like that, information being distributed through radio because we have a radio station. I think if ever there was something showed on TV how these people can be treated then it will reach them and it will get better.* ***– Lay counsellor (LCMS3)***

4.1.3 *Psycho-education and psychosocial rehabilitation for family members and service users*

Psycho-education for family members to assist them to cope better with a PWMI in their care as well as psychosocial rehabilitation for service users to assist them to understand and cope better with their illness and the effects of stigmatization and discrimination were highlighted by participants.

*Counselling and we can also give them management (techniques) … because you educate the family about the client’s condition and the client also has to have an idea what disorder she has and how she can manage it.* ***– Nurse (N4)***

4.2 ***Acceptance and support***

The participants of this study were of the opinion that helping people (family members, friends and community members) accept PWMIs would assist in reducing stigma. Acceptance should be expressed in the quality of care and support given to PWMIs and education is an important tool to achieve this.

*If the patient could be accepted and understood by the community he would have courage to do something about his life but the more he (is) teased (the more) he might be stressed out and relapses.* ***– Auxiliary social worker (ASW2)***

Support groups with members going through the same experience were seen as means of helping PWMIs to gain social and emotional support. Such support groups would provide service users the opportunity to share their experiences and perhaps learn from one another.

*Another extra option would be support groups whereby you’ll be sitting with people who are having the same challenges and talking about the same issues as well. Socially they do need support.* ***– Nurse (N9)***

4.3 ***Supervision of health care providers***

Supervision was suggested as a means of checking the activities of health care service providers to ensure they are delivering on their duties. This is expressed in the statement from one service user below.

*These people don’t treat us well- these people- these sisters they don’t treat us well… maybe there should be people to come check every month or every two weeks to see how we are being treated- to see what is happening. –****Service user (DH9)***

4.4 ***Integration at health facilities***

The current approach of integrated chronic disease management in South Africa was also perceived as a means of reducing the stigmatization of those with mental illness. This ensures that the identity of a PWMI is kept confidential and reduces stigma and discrimination as expressed by one lay counsellor.

*See what is going on here today… (you) see a diabetic, asthmatic and HIV positive person in the same line - they are all going to the same Sister for medication and confidentiality exists. On the outside it’s a cover they are the same, in some clinics before it used to be different.* ***– Lay counsellor (LCO1)***

4.5 ***Sanctions/legal action against agents of discrimination***

Sanctioning those who ill-treat PWMIs and bringing them to face the law was expressed as a means of curbing stigma was suggested by some participants. These participants were of the view that people in the society should not just get away with victimizing and abusing PWMIs.

*I think charges should be pressed on people who ill-treat mentally ill patients. –****Service user (S2)***

## Discussion

### Experiences of stigma

The results of this study show that various forms of externalized stigma are being experienced by service users from primary health facilities and from family members, neighbours, friends, church members and the general community. In relation to the different forms of stigma experienced, PWMIs reported internalized/self-stigma and recounted their experiences of externalised/experienced stigma. With regard to the latter, intensive and challenging experiences of being abused or mistreated in their families, communities and at health facilities were reported and supported by health care service providers.

### Causes of stigma

In relation to the causes of psychiatric stigma, beliefs and traditional explanatory model of mental illness play an important role in perpetuating stigma and discrimination. For example, the belief that PWMIs are pretending to be ill or are ill because they are weak is common in the community. There are also traditional beliefs that PWMIs must have been bewitched. These beliefs impact negatively on PWMIs through social isolation; neglect and maltreatment; delayed medical help-seeking and being ignored by health care staff. These experiences can further deepen a person’s mental illness through the development of depressive symptoms and low self-esteem as has been found by other studies [9,19].

Particularly worrying is that many of these beliefs about the causes of mental illness were also held by health care staff. As has been previously found, medical education does not guarantee a reduction in stigmatizing beliefs [39]. However, as asserted by Lawrie [23], the attitude of health care service providers is key to efforts to reduce psychiatric stigma and to promoting recovery in PWMI. In this study, experiences of stigma and discrimination were found to deter service users from seeking medical treatment. Within the context of the shift towards decentralized mental health care in South Africa as contained within the Mental Health Care Act of 2002 [40] and new Mental Health Care Policy Framework [41], care for people with stabilized chronic mental conditions has been decentralized to their nearest primary health care facilities. In addition to non-adherence being a product of medication not routinely being available at the PHC clinics as well as a lack of psychosocial rehabilitation services at a community level [30], non-adherence as a result of patients being unwilling to visit PHC facilities to collect their follow-up medication for fear of stigma and discrimination at the hands of the PHC staff is of added concern. This can compromise symptom management and jeopardize the shift to decentralized care, with patients who relapse needing to be re-admitted and perpetuating the revolving door phenomenon.

Also in relation to the causes of stigma and discrimination, the findings of this study suggest that the origins lie with caveats in knowledge and traditional beliefs about the causes of mental illness that can result in human rights abuses of PWMIs and delay medical help seeking. This supports the work of Thornicroft et al. [3] who posit that the stigma of PWMIs is a combination of problems relating to knowledge, attitudes and behaviour. The suggestion by Pinfold et al. [1] that interventions to reduce stigma address these knowledge caveats as well as stigmatizing beliefs and attitudes is thrown into sharp relief. It must be noted that while there is need to respect the traditional cultural beliefs of a community, there is also a need for education on the biomedical causes and treatment of severe mental illness in order to curtail stigmatizing practices which only worsen the mental health of PWMIs in the society.

### Impact of stigma on service users

As described by various authors, psychiatric stigma affects the health and well-being of PWMIs and reduces their rate of recovery [1,12,19]. Results from this study also support this stance as there were indications of how stigma and discrimination impacts negatively on the mental health of service users and marginalizes them from society as it inhibits their capacity to lead normal lives. Stigma is also a major barrier to health care and good quality of life [6,19].

### Interventions to combat stigma

Concerning possible interventions to address stigma and discrimination amongst health care staff, community and family members, the need for education and training was again emphasized [42,43]. For health care service providers, international evidence suggests the effectiveness of the integrated chronic care model [44,45] in improving health care service delivery in PHCs. In the research site in South Africa, primary health care nurses and doctors are being trained to diagnose mental illness and equipped with the knowledge and skills they need to attend to PWMIs using Primary Care (PC) 101 [46], which is an integrated set of chronic care guidelines. These guidelines, however, do not provide education on the causes of mental illness or address prevailing traditional beliefs or fears providers may have of mental illness. They also do not orientate primary health care providers on the importance of mental health to overall health [47] and thus the need for treating mental illness for improved overall health. Also, PC101 does not adequately address stigma and discrimination which has proven to be a serious barrier to the health and well-being of service users especially.

Within the context of the social contact hypothesis to reduce stigma and discrimination [48], the shift towards the Integrated Chronic Disease Management model in South Africa, whereby all service users with a chronic illness, including PWMIs, are seen at a service delivery point, should assist to reduce stigma and discrimination of PWMIs at the Primary Health Care (PHC) facility level as specified by the World Health Organization [49]. The social contact theory stems from Allport’s inter-group contact theory which aims at reducing prejudice based on ethnicity or race but it has since been extended to other stigmatizing phenomena and contexts [50]. Contact with PWMIs has been described as an effective strategy for stigma reduction [9,42]. However, contact by itself may not be effective for stigma perpetuated by health care service providers and in health care facilities as observed in this study. The effectiveness of contact in reducing prejudice has been contended [50]. In this study, contact with PWMIs when in a state where they are aggressive and needing treatment seemingly increases stigma and discriminatory practices. In the absence of sufficient education and training as well as structural support to deal with aggressive patients such as ‘safe rooms’, sedative drugs and transport to transfer such patients to the nearest outpatient facility, the power differentials between service providers and service users may create an environment which does not make ‘contact’ an appropriate strategy for stigma reduction among health care service providers. Stigma has been described as a social process rooted in social power relations [16]. As noted by Kleintjes, Fick, Railon, Lund, Malteno and Robertson [50], the characteristics of the contact setting as well as the groups and individuals involved are among the many factors that can determine the success of social contact in reducing intergroup prejudice. For contact to be successful at the PHC clinic level, education of service providers and an enabling context is key in order to address stigma at this level.

With respect to possible interventions to address stigma and discrimination amongst family and community members, this study suggests the need for psycho-education of family and community members of the causes and symptoms of mental disorders as well as how to care for PWMIs [51]. In the context of deinstitutionalization, there has been an increased clamour internationally and in South Africa for the involvement of family members in the treatment of the mentally ill in order to improve their health outcomes [39]. Psycho-education and psychosocial rehabilitation programmes for PWMIs are important not only for the enhancement of their mental health and well-being but for aiding their recovery process and empowerment [52].

Having support groups for service users as suggested by a nurse in this study, may also be an important strategy mobilising service users towards advocacy. In addition to being beneficial to their mental health, greater involvement of service users in awareness raising and other advocacy programmes in South Africa may assist in breaking down stigmatizing and discriminatory attitudes of the public and health care providers [53]. This will also increase service uptake and re-integration of persons with mental disorder into the society.

#### Recommendations

Suggestions to reduce stigma have centred on training, education, contact, mass media campaigns and a broad range of multifaceted interventions. The implications of the findings in this study for stigma reduction interventions are discussed below.

1. Psycho-educational interventions should be mounted with PHC staff to address the myths and traditional beliefs on the causes of mental illness which influence medical stigma. In addition, it would be important for PHC staff to understand the relationship between mental health and physical health and the importance of identification and treatment of mental illness to health care as a whole. Testimonials of PWMI who have recovered as a result of treatment would be useful in this regard.

2. There is need for PHC facilities to be adequately equipped and their staff trained on how to handle patients with severe mental illness who may require restraining.

3. Media campaigns and interventions to create awareness of the causes of mental illness and effective treatments as well as the need for supportive community environments should be designed to reduce stigma and discrimination in communities. In particular, involvement of people with severe mental illness in recovery may make inroads towards changing stereotypes that they are violent and destructive.

4. Psychoeducation programmes for families and caregivers as well as support for caring for PWMI is needed. Such programmes should be tailored to the specific impairments and challenges linked with each mental illness.

5. Group-based psychosocial rehabilitation programmes for service users with severe mental illness should be made available within the community. These should include empowering service users to deal positively with stigmatizing and discriminatory behaviours and maintain their own psychosocial wellbeing to reduce the impact of externalised/experienced and internalized stigma. They should also provide opportunities for positive social interactions between service users and the wider community, for example through work activity.

6. Advocacy interventions on the part of PWMI would be important to assist in showing how PWMI are not a threat to society and can make a significant contribution to the development of policy and services.

#### Limitations of the study

This study did not seek to explore the differences in attitude and conceptualization of mental illness in this context as compared with a westernized definition of mental illness. It also did not explore traditional conceptualization of psychiatric stigma within the cultural context of the study participants. Though this may serve as a possible bias to this study, the interview schedule used to guide data collection (as described in data collection section) had questions which all participants could relate to and which elicited responses that speak to stigma and discrimination as understood by the participants. The participants interviewed did not include medical doctors and psychiatrists who deal with PWMIs. Most PHC facilities in South Africa are not staffed with a permanent medical doctor or psychiatrist. This poses a problem with the quality of mental health care available at the PHC centres. For the service users with schizophrenia, there may have been some response bias because interviews were conducted in the Mental Health Society offices or in the clinic and service users may not have felt comfortable to disclose certain experiences in these settings. Also, this is a qualitative study involving three clinics in a specific context therefore the generalizability of the findings and applicability of the recommendations based on the findings is limited. The use of convenient sampling may also have introduced bias to the findings.

## Conclusion and implication for interventions to reduce stigma

This study highlights that stigma and discrimination in its broad forms of internalised (self-stigma) and externalized/experienced or public stigma [10] are rife in the lives of service users interviewed and negatively affects their health status and chances of recovery.

In the context of decentralization and the shift to integration of mental health services into primary health care in low- and middle-income countries and in South Africa particularly, the need for anti-psychiatric stigma interventions is foregrounded. As has been shown by this study and others, stigma and discrimination of people with mental illness is rife in PHC settings and can result in delayed help-seeking and non-adherence due to reluctance to attend PHC facilities for ongoing care.

The need for interventions at all levels is also highlighted to promote more supportive home and community environments.

### Suggestion for further research

The authors recommend an exploration of the conceptualization of stigma in this setting. Also, the use of quantitative measures to assess the level of stigma will be a useful tool in monitoring interventions and time trends.

## Competing interests

The authors declare that they have no competing interests.

## Authors’ contribution

IP and COE were involved with the design of the study. Data collection was done by CB, TK and OS. The analysis of data was carried out by COE, CB, TK, OS and IP. COE wrote the first draft of the manuscript while IP, GT, CB, TK and OS were involved in critical review of the initial manuscripts. All authors read, edited and approved the final manuscript.

## Pre-publication history

The pre-publication history for this paper can be accessed here:

http://www.biomedcentral.com/1471-244X/14/191/prepub
